# From Crowd to Care: Telemedical Delegation in Mass Gathering Medicine and Its Projected Impact on Hospital Transport—A Modified Delphi Study with Retrospective Modelling

**DOI:** 10.3390/healthcare14183095

**Published:** 2026-09-20

**Authors:** Robert Arimond, Michael Czaplik, Jan Larmann, Hanna Schröder, Anna Müller, Andreas Follmann

**Affiliations:** 1 Faculty of Medicine, RWTH Aachen University, 52074 Aachen, Germany; mczaplik@ukaachen.de (M.C.); jlarmann@ukaachen.de (J.L.); hschroeder@ukaachen.de (H.S.); anna.mueller@ukaachen.de (A.M.); afollmann@ukaachen.de (A.F.); 2 Docs in Clouds Telecare GmbH, 52074 Aachen, Germany

**Keywords:** telemedicine, emergency medicine, mass gathering medicine, task delegation, remote consultation

## Abstract

**Background/Objectives**: Physician shortages increasingly challenge the provision of medical care at mass gathering events. Telemedicine may expand the treatment capabilities of Emergency medical technician intermediates (EMT-I), but the extent to which medical procedures are suitable for telemedical delegation and how such a care model might affect hospital transport rates remain unclear. **Methods**: In this retrospective modelling study, patient records from the 2022 Summerjam Festival were reviewed, and all documented diagnostic and therapeutic measures were extracted for assessment in a modified Delphi process. We then applied the resulting expert consensus on telemedical delegability to the original patient cohort to classify cases by theoretical suitability for telemedical management and to project hospital transport rates under a predefined telemedicine-only scenario. **Results**: Based on the retrospective application of the final modified Delphi consensus, 385 of 403 cases (95.5%; 95% CI 93.0–97.3%) were classified as theoretically eligible for telemedicine-only management. Overall, 144 of 200 interventions were classified as fully delegable, 48 as suitable for bridging delegation, and 8 as requiring an on-site physician. Under the predefined telemedicine-only scenario, the projected hospital transport rate increased from 6.2% to 9.2%, corresponding to an absolute increase of 3.0 percentage points (95% CI 1.3–5.0%). **Conclusions**: These findings suggest that telemedicine may substantially expand the theoretical scope of care provided by EMT-Is in mass gathering medicine. The projected increase in hospital transports under the predefined telemedicine-only scenario was partly determined by the assumption that patients requiring bridging or physician-only measures would require hospital transport in the absence of an on-site physician. Hybrid care models combining telemedical physician support with targeted on-site physician availability may therefore warrant prospective evaluation in future mass gathering settings.

## 1. Introduction

### 1.1. Mass Gathering Events and Mass Gathering Medicine

Mass gathering events (MGEs) are defined as planned or spontaneous events involving a large number of participants [1,2]. Such events can place considerable strain on local healthcare systems and therefore require additional medical resources and appropriate planning for on-site medical care [3]. In Germany, mass gathering medicine (MGM) is typically provided by disaster response organizations as a private-sector service. As a result, the personnel involved largely overlap with those working in disaster medicine [4,5]. Depending on the event and risk assessment, MGM services may be staffed by physicians, emergency medical technician professionals (EMT-Ps), emergency medical technician intermediates (EMT-Is), and personnel with lower levels of qualification [6].

Most patients presenting to MGM services can be treated by non-physician personnel. However, some require diagnostic or therapeutic interventions that exceed the competencies of EMT-Is [7]. Physician involvement therefore remains an important part of comprehensive medical care at MGEs, even though physician involvement is relatively infrequent [8].

In the terminology used in this study, EMT-I refers to the German qualification “Rettungssanitäter”. In North Rhine-Westphalia, where the investigated event took place, this qualification requires at least 520 h of theoretical, clinical, and practical training and qualifies personnel for different functions in non-emergency patient transport, emergency medical services, and civil protection [9]. In contrast, the German “Notfallsanitäter”, referred to as EMT-P in this study, completes a three-year state-regulated professional training program comprising at least 4600 h of theoretical, clinical and practical training [10,11]. Their professional competencies include independent patient assessment and emergency care as well as, under defined legal conditions, independently performed invasive and pharmacological interventions [10]. EMT-Is and EMT-Ps therefore differ substantially in their level of training and scope of independent practice. Neither category should be considered directly equivalent to similarly named emergency medical professions in other healthcare systems, where training requirements and scopes of practice may differ. These differences in baseline competencies should be considered when transferring the findings of this study to other healthcare systems.

### 1.2. Physician Shortage and Telemedicine as a Potential Solution

At the same time, providing physician coverage for MGEs is becoming increasingly difficult. Like many other countries, Germany is facing a physician shortage [12], making it harder to recruit physicians for MGM services. Similar difficulties have been reported in on-call medical services and emergency clinics [13].

Telemedicine may offer a way to address this challenge. It is defined as the use of telecommunications technology to deliver healthcare services and medical information remotely [14]. By enabling physicians to support on-site personnel from a distance, telemedicine may expand the range of available treatment options. Previous studies have shown that urgent medical measures are not delayed when telemedicine is used [15].

Telemedicine is already established in prehospital emergency care and out-of-hours medical services [13,16]. Previous studies have reported the use of telemedical physician support to facilitate advanced medical care by appropriately trained emergency medical personnel beyond their usual scope of practice.

Telemedical approaches have also been implemented in different mass gathering settings. At the Summerjam Festival in Germany, telemedical physician support was prospectively implemented to enable physician-guided treatment by on-site emergency medical personnel [17]. Internationally, the Hajj pilgrimage in Saudi Arabia represents a notably demanding mass gathering setting, with a substantial and different medical burden among pilgrims [18]. More recently, virtual care has also been implemented in this setting. During the 2024 Hajj season, an Emergency Medicine and Rapid Response Virtual Team complemented on-site medical teams and emergency departments, while dedicated telemedical services, including telestroke care, have been provided during the 2023 and 2024 Hajj seasons [19,20]. These reports demonstrate that telemedicine has been implemented in mass gathering settings for different purposes, ranging from support of on-site treatment to virtual emergency and specialist consultation. However, the available evidence remains limited and heterogeneous, and the organizational models, target populations, personnel qualifications, and clinical objectives differ substantially between settings.

Most MGM services in Germany are staffed primarily by EMT-Is rather than EMT-Ps. Whether telemedical delegation could similarly expand the theoretical treatment options available to EMT-Is in the MGM setting remains unclear. Without physician support, limitations on delegated measures may also affect the need for hospital transport [21].

### 1.3. Aim of the Study

Against this background, this study examined the theoretical potential of telemedical physician support in MGM, using the 2022 Summerjam Festival as an example. First, a modified Delphi process was conducted to classify medical measures according to their potential delegability to EMT-Is with telemedical physician support. The resulting expert consensus was subsequently applied retrospectively to patient data from the event to estimate the proportion of cases theoretically manageable within such a care model and to project its potential effect on hospital transport rates.

The primary objective was to determine the proportion of cases that would be theoretically manageable by EMT-Is with telemedical physician support while retaining access to all conventionally available measures. At the time of study planning, a benchmark of 79.5% was derived from previously published data from the Aachen tele-EMS system. Following implementation of this system, the proportion of conventional EMS missions involving an on-site emergency physician had decreased to 20.5%; accordingly, 79.5% of EMS missions were conducted without an on-site emergency physician [22]. No directly comparable mass gathering cohort was available at the time of study planning in which telemedical physician support had been implemented and its association with hospital transport rates systematically evaluated. The Aachen data were therefore selected as a pragmatic external reference and, importantly, covered the overall EMS population rather than a subgroup restricted to specific or potentially life-threatening emergencies. However, given the substantial differences between conventional EMS populations and patients presenting at mass gathering events, this benchmark was used for contextual comparison and should not be interpreted as implying similarity between these populations. In addition, the study evaluated the projected change in hospital transports under the predefined telemedicine-only scenario compared with the hospital transport rate actually observed during the event.

## 2. Materials and Methods

### 2.1. Study Design and Data Source

This study consisted of two consecutive phases. First, a modified Delphi process was conducted to classify diagnostic and therapeutic measures according to their potential delegability to emergency medical technician intermediates (EMT-Is) with telemedical physician support in the context of mass gathering medicine (MGM). Second, the resulting consensus was retrospectively applied to patient records from the 2022 Summerjam Festival to estimate the theoretical applicability of telemedical delegation at the case level and to project hospital transport rates under a predefined telemedicine-only scenario.

Patient records originated from the Summerjam Festival 2022, a three-day reggae music festival held in Cologne, Germany, with approximately 30,000 attendees. During the event, all medical consultations were managed through a central medical station. At least one emergency medical technician professional (EMT-P) and one physician were continuously present at the medical station. The physician also served as the tele-emergency physician for telemedical consultations performed during the event [17]. Staffing levels and transport resources varied according to the anticipated workload over the course of the festival. Detailed time-dependent numbers and qualifications of additional personnel and available transport resources were not systematically recorded.

Telemedicine was implemented within the medical service as part of a prospective feasibility study [17]. However, the present study did not evaluate the actual telemedical consultations performed during the event. Instead, all documented patient encounters were retrospectively analyzed to assess the theoretical applicability of telemedical delegation based on the modified Delphi consensus.

A total of 404 patient encounters had been documented during the event. One case was excluded because essential information required for analysis was missing. The remaining 403 cases were included in the study.

All treatments were documented using standardized patient care records containing information on medical history, vital signs, diagnosis, injuries, diagnostic procedures, therapeutic interventions, administered medications including dosages, and patient disposition. During routine event operations, record completeness was not actively monitored. All records were anonymized and digitized in Microsoft Excel (Microsoft 365, Microsoft Corp., Redmond, WA, USA) before analysis.

### 2.2. Case Preparation and Categorization

One investigator (RA) reviewed all eligible patient records and extracted all documented diagnostic procedures, therapeutic interventions, and administered medications. This process resulted in 200 context-based distinct diagnostic and therapeutic measures that were subsequently submitted for assessment in the modified Delphi process.

To facilitate expert evaluation, cases were assigned to clinically meaningful diagnostic categories based on the documented presenting complaint, working diagnosis, and treatment requirements. The categorization broadly followed the diagnostic structure used in German emergency medical service documentation systems compatible with the DIVI documentation framework. Categories included abdominal complaints, allergic reactions and skin conditions, circulatory problems, headaches, wounds and surgical conditions, substance-related conditions, and critical conditions.

Cases were classified as critical conditions if they fulfilled German trauma or non-trauma resuscitation room criteria [23,24]. Classification was based on the documented patient history, presenting condition, and recorded vital signs.

### 2.3. Modified Delphi Process

The modified Delphi process was intended to determine which context-based diagnostic and therapeutic measures could potentially be delegated to an EMT-I acting as a tele-paramedic and which measures would require direct physician involvement.

Experts were asked the following question for each measure: “In case of (…),

- would you delegate the measure to the tele-paramedic without calling an emergency physician to the scene (telemedicine only),
- would you delegate the measure and simultaneously request an emergency physician as bridging treatment, or
- would you not delegate the measure at all (physician treatment only)?”

In accordance with previous Delphi studies, consensus was predefined as agreement by at least 80% of participating experts. Measures reaching consensus were not reassessed in subsequent rounds. Experts were instructed to assume that the respective intervention was clinically indicated and that the administered dose was appropriate for the individual case. Furthermore, experts were informed that telemedical supervision included explanation of procedures whenever necessary.

The modified Delphi process consisted of three stages. In the first round, all identified measures were grouped according to the diagnostic categories described above and assessed using an anonymous online survey conducted over a three-week period. The invitation was distributed to eligible physicians at the Aachen Institute for Rescue Management and Public Safety (ARS). Eligibility required qualification as a tele-emergency physician, a background in anesthesiology, fulfillment of the applicable qualification requirements for tele-emergency medical practice, and experience in mass gathering medicine. The exact number of individuals who received the invitation was not documented. Because the study required highly specialized physicians with expertise in both tele-emergency medicine and mass gathering medicine, a purposive expert sample was used. A minimum of five completed questionnaires was predefined for progression to the second round. Detailed individual data on years of professional experience in emergency medicine, telemedicine, and mass gathering medicine were not systematically collected.

In the second round, the results of the first round were presented in a second anonymous online survey to physicians meeting the same eligibility criteria. The exact number of individuals who received the invitation was not documented. Because participation in the online Delphi rounds was anonymous, individual participation could not be tracked across rounds. Consequently, it could not be determined whether the same experts participated in both rounds. The second-round survey was available for one week.

Measures that did not achieve consensus after the second round were discussed during a one-day online expert workshop with participants meeting the same eligibility criteria as in the preceding rounds. In contrast to the previous rounds, participants in the workshop were not anonymous to one another. Each of the remaining measures was discussed individually until a final classification could be put to a vote. The predefined consensus threshold of ≥80% agreement was retained for the workshop. Measures that still failed to reach consensus after workshop discussion were classified as “no consensus”.

Tele-emergency physicians in Germany are board-certified physicians who have completed at least 500 conventional prehospital emergency missions and additional qualification requirements for telemedical emergency care [25].

### 2.4. Application of Delphi Results and Definition of the Transport Scenario

Following completion of the modified Delphi process, the consensus classifications were applied retrospectively to all included patient encounters.

Measures were classified into four categories: fully delegable through telemedicine, delegable as bridging treatment until arrival of an emergency physician, physician-only measures, and measures without consensus.

For the case-level analysis, a predefined conservative telemedicine-only scenario was modelled in which no physician was assumed to be physically available on site. Cases were classified as eligible for telemedicine-only management only if all documented diagnostic and therapeutic measures were considered fully delegable through telemedicine. If at least one required measure was classified as physician-only or as bridging treatment requiring physician attendance, the case was classified as requiring hospital transport. This assumption reflected a model in which patients requiring an intervention that could not be completed by telemedical delegation alone would require transport to a physician-staffed hospital in the absence of an on-site physician. Encounters that had actually resulted in hospital transport during the event remained classified as requiring transport in the modelled scenario.

### 2.5. Outcomes

The primary outcome was the proportion of patient encounters theoretically eligible for telemedicine-only management according to the modified Delphi consensus.

A benchmark of 79.5%, derived from previously published data from the Aachen tele-EMS system [22], was used as a contextual external reference. The benchmark represented the complementary proportion of EMS missions not involving an on-site emergency physician after implementation of the tele-EMS system and was not treated as a directly comparable control population.

The secondary outcome was the projected hospital transport rate under the predefined telemedicine-only scenario compared with the hospital transport rate actually observed during the event. Clinical outcomes, adverse events, diagnostic correctness, and the safety or clinical effectiveness of actual telemedical care were not assessed.

### 2.6. Statistical Analysis

All statistical analyses were performed using IBM SPSS Statistics versions 28.0 and 32.0 (IBM Corp., Armonk, NY, USA). Exact 95% confidence intervals were calculated using the Clopper-Pearson method for single proportions, whereas 95% confidence intervals for differences between paired proportions were calculated using the Newcombe method.

The primary outcome was analyzed descriptively and is reported with its exact 95% confidence interval. The predefined external benchmark of 79.5% was used for contextual comparison only; no formal hypothesis test against this benchmark was performed.

For the secondary outcome, the hospital transport status actually observed during the event was compared descriptively with the projected transport status under the predefined telemedicine-only scenario. Absolute differences in paired proportions are reported together with their corresponding 95% Newcombe confidence intervals. No formal hypothesis test was performed for this comparison.

## 3. Results

### 3.1. Modified Delphi Results

A total of 403 patient encounters were available for analysis. From these cases, 200 context-based diagnostic and therapeutic measures were identified and submitted for modified Delphi assessment.

Five experts participated in the first modified Delphi round. Consensus was achieved for 138 of the 200 measures (69.0%). Of these, 131 measures were classified as fully delegable through telemedicine, while seven measures were considered suitable for delegation as bridging treatment until a physician arrives. No consensus was reached for the remaining 62 measures.

Five experts participated in the second modified Delphi round. Consensus was achieved for an additional 26 measures. Thirteen of these were classified as fully delegable and thirteen as suitable for bridging delegation. After two modified Delphi rounds, consensus had therefore been reached for 164 of 200 measures (82.0%). Thirty-six measures remained unresolved and were subsequently discussed during the expert workshop.

Four experts participated in the third modified Delphi stage. Each of the 36 remaining measures was discussed individually and subsequently put to a vote. As the predefined consensus threshold of ≥80% was retained, unanimous agreement (4/4; 100%) was required for consensus in the workshop. All 36 measures reached unanimous agreement. Of the 36 measures discussed during the workshop, 28 were classified as suitable for bridging delegation and eight were classified as physician-only measures. The level of agreement for each individual measure, expressed as n/N and percentage, together with the stage at which consensus was reached, is provided in Supplementary Table S1.

**Figure 1:**
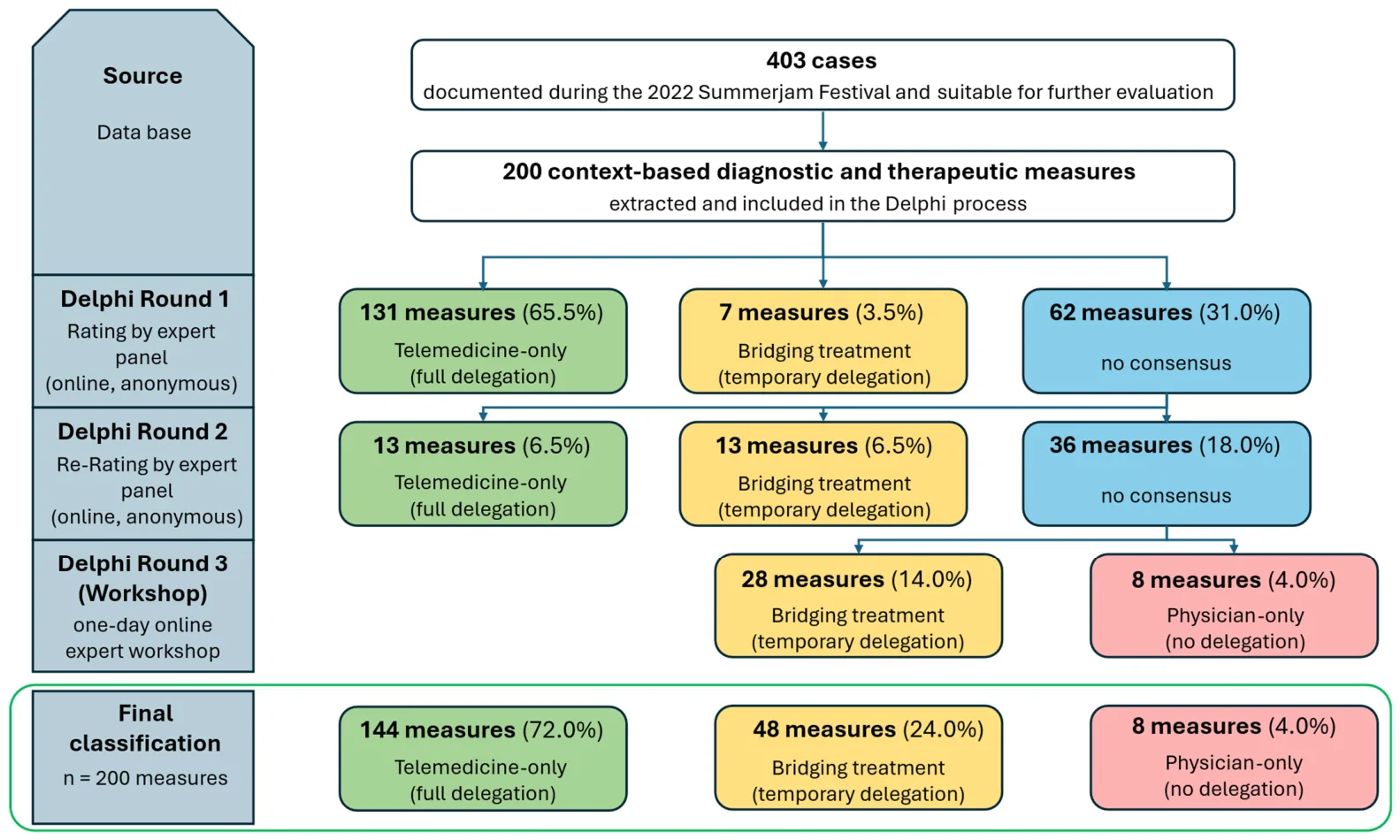
Study workflow and modified Delphi consensus process for classifying diagnostic and therapeutic measures according to telemedical delegability. The green border presents how the 200 measures are categorized, based on the consensus, into “full delegation,” “temporary delegation,” and “no delegation”.

Overall, 144 of the 200 assessed measures (72.0%) were considered fully delegable through telemedicine, 48 measures (24.0%) were considered suitable for bridging delegation, and eight measures (4.0%) required direct physician involvement (see Figure 1).

Most diagnostic procedures, physical examination techniques, oral medications, intravenous access procedures, and several intravenous medications were considered fully delegable. Measures requiring physician attendance primarily included the administration of epinephrine outside cardiac arrest, the administration of fentanyl, and the use of tissue adhesive for wound closure (see Supplementary Table S1). All life-saving interventions assessed in critical conditions were classified as suitable for bridging delegation but required subsequent direct physician involvement.

### 3.2. Primary Outcome: Theoretical Eligibility for Telemedicine-Only Management

Of the 403 included patient encounters, 385 (95.5%; 95% CI 93.0–97.3%) were classified as theoretically eligible for telemedicine-only management according to the modified Delphi consensus. For contextual comparison, the predefined external benchmark derived from the Aachen tele-EMS system was 79.5%.

An additional 14 cases (3.5%; 95% CI 1.9–5.8%) required at least one measure classified as bridging treatment. Consequently, 399 of 403 cases (99.0%) were classified as amenable to some form of telemedical delegation, either as telemedicine-only management or as bridging treatment until direct physician care became available.

**Figure 2:**
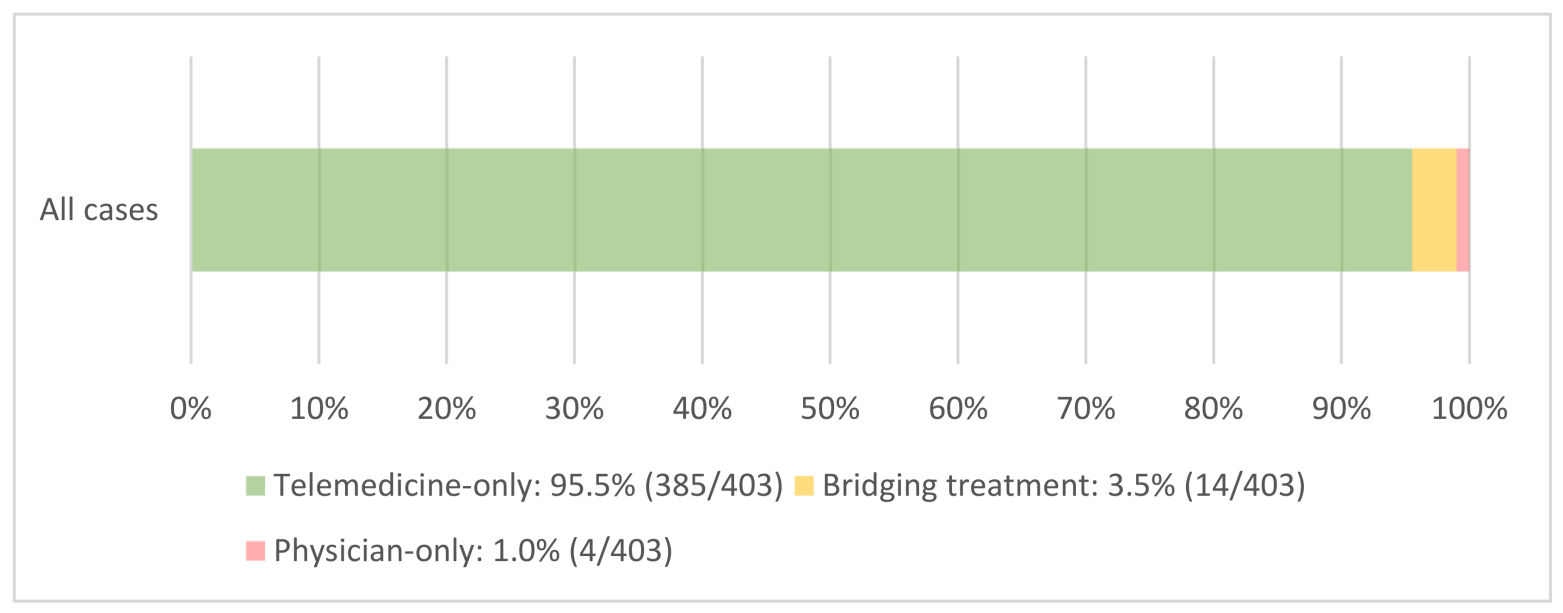
Final classification of patient encounters according to theoretical eligibility for telemedical delegation in the study cohort.

Four cases (1.0%; 95% CI 0.3–2.5%) required at least one physician-only measure and were therefore not amenable to telemedical delegation (see Figure 2).

The 18 encounters not classified as theoretically eligible for telemedicine-only management comprised 14 cases requiring bridging treatment and 4 cases requiring physician-only treatment. The bridging cases included six anaphylactic or allergic reactions, three seizures, three presentations involving syncope or chest pain, one acute asthma exacerbation, and one case of epistaxis associated with headache and nausea. The four physician-only cases involved traumatic wounds treated with tissue adhesive. Of these 18 encounters, 6 had actually resulted in hospital transport during the event, whereas 12 had been managed without hospital transport. Presenting conditions, treatments actually performed, and actual hospital transport status are summarized in Table 1.

### 3.3. Secondary Outcome: Hospital Transport Rate

**Figure 3:**
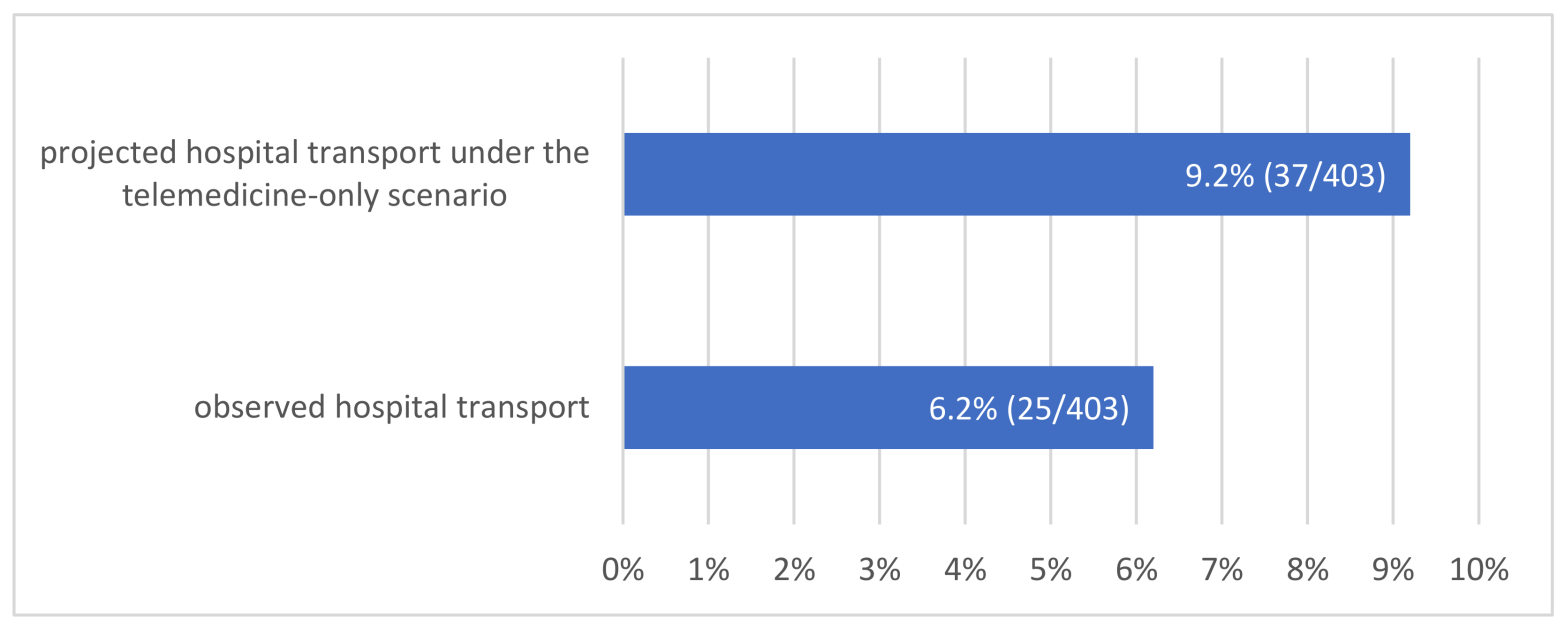
Comparison of the observed hospital transport rate and the projected hospital transport rate under the predefined telemedicine-only scenario.

During the event, 25 of 403 patients (6.2%) were transported to hospital. In the projected telemedicine-only scenario, 37 of 403 patients (9.2%) were classified as requiring hospital transport. Of the 18 encounters not classified as theoretically eligible for telemedicine-only management, 6 had already resulted in hospital transport during the event and therefore did not contribute additional transports in the projected scenario. The remaining 12 encounters had originally been managed without hospital transport but were classified as requiring transport under the predefined telemedicine-only scenario. All patients who had been transported during the event remained classified as requiring hospital transport in the projected telemedicine-only scenario (see Figure 3).

Overall, the projected telemedicine-only scenario resulted in a modelled absolute increase in the hospital transport rate of 3.0 percentage points (95% CI 1.3–5.0 percentage points), from an observed rate of 6.2% to a projected rate of 9.2%.

## 4. Discussion

The present study evaluated the potential role of telemedicine in mass gathering medicine (MGM) by assessing the expert-perceived delegability of diagnostic and therapeutic measures to EMT-Is and applying the resulting modified Delphi consensus to a real-world festival cohort. Of the 200 assessed measures, only 8 required direct physician involvement, while 192 measures were considered either fully delegable or suitable for delegation as bridging treatment. When applied to the Summerjam Festival cohort, 95.5% of cases were classified as theoretically eligible for telemedicine-only management. Under the predefined telemedicine-only scenario, the projected hospital transport rate increased from the observed rate of 6.2% to 9.2%. These data describe the theoretical scope of telemedical delegation under the model’s assumptions and should not be interpreted as evidence of clinical feasibility, safety, or effectiveness.

### 4.1. Delegability of Medical Measures

The high proportion of delegable measures is consistent with previous findings describing the use of telemedicine in prehospital emergency care and MGM [15,17,25]. Most diagnostic procedures, physical examination techniques, medication administrations, and routine therapeutic interventions were considered suitable for telemedical delegation. Previous studies have reported that telemedical physician support can facilitate advanced medical care by appropriately trained non-physician personnel [15]. However, the present study broadens these observations only at the level of expert-perceived delegability and did not prospectively assess whether the measures classified as delegable could be performed safely or effectively by EMT-Is in the investigated setting.

Remarkably noteworthy is the small number of measures that were considered completely non-delegable. Only the administration of epinephrine outside resuscitation scenarios, the administration of fentanyl, and the application of tissue adhesive for wound closure were consistently classified as physician-only procedures. Even in critically ill patients, experts agreed that life-saving interventions could be initiated through telemedical delegation as emergency bridging measures, provided that physician attendance was subsequently ensured. This distinction is clinically important, as telemedicine would not be intended to replace direct physician care in critically ill patients. Rather, telemedical delegation may allow trained on-site personnel to initiate time-critical interventions immediately as direct physician support is simultaneously being mobilized. Previous studies have reported that telemedical physician support did not delay the delivery of time-critical interventions and was not associated with worse patient outcomes [15,17]. While these findings supply relevant clinical context for the present expert assessments, the current study did not evaluate these outcomes and therefore cannot confirm their pertinence to telemedical delegation to EMT-Is in MGM.

The high level of agreement regarding medication delegation is also consistent with existing telemedicine literature. Previous studies in prehospital emergency care have reported the telemedical delegation of various medication classes, including crystalloid fluids, analgesics, bronchodilators, glucose, antiemetics, sedatives, vasopressors, and anticonvulsants [26]. The present findings largely reflect these experiences and suggest that similar principles may be applicable to the MGM setting. However, the modified Delphi consensus represents expert judgment and cannot establish the clinical safety of medication delegation to EMT-Is in this setting.

Importantly, the telemedical model evaluated in this study differs substantially from conventional remote prescribing. Concerns regarding telemedical medication prescribing often arise when physicians have limited opportunities to independently assess patients and verify diagnoses [27]. In contrast, the model investigated in the present study assumes that patients are assessed directly on site by trained medical personnel, including structured history taking, physical examination, and assessment of vital signs. Telemedical physicians would therefore base their decisions on considerably more clinical information than is typically available during remote outpatient consultations.

Furthermore, medication administration within MGM would occur in a supervised medical environment and would generally be restricted to the duration of treatment within the medical station. This setting would allow immediate review of treatment effects and adverse events. The broad delegability of medications observed in this study should therefore not be interpreted as unrestricted telemedical prescribing but rather as expert-perceived suitability for telemedical supervision of medication use within a controlled clinical environment. The present study did not assess medication-related adverse events, diagnostic errors, clinical deterioration, or treatment outcomes.

Overall, medication delegation itself was rarely identified by the expert panel as an absolute barrier to telemedical delegation. Instead, the modelled necessity for direct physician involvement was concentrated in a comparatively small subset of measures and clinical presentations.

### 4.2. Interpretation of the Increased Transport Rate

Under the predefined telemedicine-only scenario, the hospital transport rate increased from the observed 6.2% to a projected 9.2%, corresponding to an absolute modelled increase of 3.0 percentage points. This finding requires interpretation in the context of the scenario definition.

Under the telemedicine-only scenario, any case requiring physician attendance according to the modified Delphi consensus was classified as requiring hospital transport. As a result, patients who might otherwise have been managed by an on-site physician were categorized as transport cases. Notably, the increase in transports was limited to 12 additional patients despite 18 cases being classified as either requiring bridging treatment or direct physician management. This occurred because six of these patients had already required hospital transport during the event and therefore did not contribute to additional transports in the projected scenario.

Consequently, the projected increase in hospital transport is partly determined by the predefined modelling assumption and should not be interpreted as evidence that telemedicine itself increases hospital transport.

Furthermore, the increase corresponds to approximately four additional transports per festival day. This study did not assess the impact of the additional transports on regional emergency medical services and hospital resources. Because the transport scenario was modelled retrospectively, actual transport decisions under a prospectively implemented telemedicine-only system could differ from these projections.

The baseline transport rate observed during the Summerjam Festival was higher than that reported for several other European music festivals [2,28]. Environmental aspects like high temperatures, prolonged event duration, and venue-specific characteristics may have contributed to this observation [29]. Direct comparisons should therefore be interpreted cautiously because event characteristics, patient populations, medical resources, and transport thresholds may differ between events.

### 4.3. Implications for Future Mass Gathering Medicine Concepts

The findings show that telemedicine may represent one potential approach to addressing physician shortages in MGM. However, the results also indicate that telemedicine should not necessarily be viewed as a complete replacement for physician presence.

Instead, future MGM concepts may benefit from hybrid models combining telemedical physician support with mobile physician response capacities. In such a model, physicians would not be permanently assigned to a medical station but could be dispatched when telemedical assessment identifies a necessity for direct physician involvement.

In the present cohort, 18 of 403 encounters (4.5%) required at least one intervention classified as suitable for bridging delegation or as physician-only treatment and would therefore have required direct physician involvement according to the modified Delphi consensus. Across the three-day festival, this corresponds descriptively to an average of six such encounters per day. Fourteen of these encounters allowed bridging delegation while awaiting direct physician care, whereas four required a physician-only intervention.

These figures provide an estimate of the potential frequency of physician activation under the assumptions of the present model rather than an observed staffing requirement. The temporal distribution of these encounters, simultaneous physician demands, response times, and the clinical consequences of delayed direct physician availability were not evaluated. The present data therefore cannot determine the number of physicians, their location, or the response times required for a hybrid staffing model.

Several additional aspects relevant to implementation were beyond the scope of the present study. These include the reliability of communication infrastructure and appropriate fallback procedures, training and credentialing requirements for on-site personnel, medicolegal responsibilities associated with telemedical delegation, patient acceptance, workload and capacity requirements of the telemedicine centre, as well as implementation costs and expandability. These factors should be specifically addressed in prospective implementation studies before broader adoption of such care models in MGM.

From a health-economic perspective, potential reductions in continuous on-site physician staffing would need to be compared against the costs of telemedical infrastructure, staffing of the telemedicine centre, and any additional ambulance transports generated by the care model. Conversely, shared telemedical physician resources supporting multiple events or locations could potentially improve resource utilization. The present study did not include a formal health-economic analysis, and whether such models result in overall cost savings therefore remains unknown.

The present findings also suggest that the projected need for additional transports was related to the absence of on-site physician availability within the predefined model rather than to limitations in EMT-I competencies alone. Whether deployment of EMT-Ps instead of EMT-Is would substantially alter projected transport rates remains unclear and should be evaluated in future studies.

### 4.4. Strengths and Limitations

A major strength of this study is the combination of a structured modified Delphi process with a real-world MGM dataset. Rather than evaluating theoretical scenarios alone, expert consensus was directly applied to documented patient encounters from a large music festival.

Several limitations should be considered. First, the dataset was derived from a single three-day music festival and may not be representative of other types of mass gatherings. Different event settings may present different patient cohorts and treatment requirements. Furthermore, the same cohort was used to derive the context-based list of measures and to assess their case-level applicability, and no independent external cohort was used for validation. These findings should therefore be regarded as exploratory and context-dependent.

Second, only a small number of critically ill patients were included, limiting the range of critical care interventions available for assessment. However, critically ill patients are generally uncommon at music festivals [8].

Third, case categorization and extraction of the documented measures were performed by a single investigator and were not independently validated, introducing the possibility of extraction and classification bias.

Fourth, the study modelled a telemedicine-only scenario based on expert consensus rather than prospectively evaluating telemedical management of all included cases. Consequently, the analysis cannot establish clinical feasibility, safety, effectiveness, diagnostic accuracy, or actual patient outcomes associated with telemedical delegation. Adverse events, diagnostic errors, and clinical deterioration were not assessed.

The projected increase in hospital transport is partly inherent to the scenario definition because encounters requiring bridging or physician-only measures were classified as requiring transport in the absence of an on-site physician. The projected transport rate should therefore not be interpreted as a causal effect of telemedicine on hospital transport.

The modified Delphi process involved a limited number of highly specialized experts. Although this reflects the restricted availability of physicians with experience in both tele-emergency medicine and MGM, larger expert panels and multicenter studies would further strengthen the evidence base. The limited panel size restricts the representativeness and may reduce the stability of the resulting consensus. The exact number of invited experts was not documented, preventing calculation of a response rate, and detailed individual data on years of experience in emergency medicine, telemedicine, and MGM were not systematically collected.

Moreover, because participation in the first two rounds was anonymous, individual participants could not be tracked across rounds. It therefore remains unknown whether the same experts participated in both rounds, which differs from the iterative reassessment by a stable panel characteristic of a classical Delphi process. The third stage was conducted as a non-anonymous expert workshop. All 36 measures remaining after the first two rounds ultimately reached unanimous agreement during this stage. Although structured discussion may facilitate clarification of differing assessments, the non-anonymous format may also introduce group dynamics, including conformity or dominance effects. These effects cannot be excluded and should be considered when interpreting the final consensus.

Finally, detailed time-dependent information on additional medical personnel and transport resources during the festival was not systematically recorded. The operational context of the event can therefore only be characterized to a limited extent, which may further restrict the transferability of the projected staffing and transport implications to other mass gathering events.

### 4.5. Future Research

Future studies should evaluate telemedicine in different MGM settings and event types to improve the generalizability of these conclusions. Prospective studies should additionally assess clinical outcomes, adverse events, diagnostic accuracy, and patient acceptance. External validation in independent event cohorts would be important to determine whether the high proportion of encounters classified as theoretically eligible for telemedicine-only management is reproducible.

In addition, prospective investigations of hybrid care models uniting telemedical support with mobile physician response concepts appear remarkably promising. Such approaches may help balance the benefits of telemedicine with the requirement for direct physician involvement in selected cases, even when physician resources are limited. Future studies of such models should also assess the timing and clustering of physician activations, required response times, communication reliability, training requirements, telemedicine-center workload and capacity, medicolegal aspects, implementation costs, scalability, and effects on emergency medical services and hospital utilization.

## 5. Conclusions

The modified Delphi results suggest that telemedical physician support may substantially expand the theoretical scope of care provided by EMT-I in mass gathering medicine. In this study, 95.5% of patient encounters were classified as theoretically eligible for telemedicine-only management, while only a small proportion of diagnostic and therapeutic measures required direct physician involvement.

However, under the predefined telemedicine-only scenario, the projected hospital transport rate increased from the observed rate of 6.2% to 9.2%. This modelled increase was partly determined by the assumption that patients requiring bridging or physician-only measures would require hospital transport in the absence of an on-site physician.

Hybrid care models combining telemedical physician support with targeted on-site physician availability may therefore represent a potential approach to MGM care but require prospective evaluation to determine their clinical safety, effectiveness, operational feasibility, and impact on hospital transport.

## Figures and Tables

**Table 1:** Characteristics of the 18 encounters not classified as theoretically eligible for telemedicine-only management.

Modified Delphi Classification	Presenting Condition	Quantity	Treatments Performed Across These Encounters	Actual Hospital Transport
Physician-only	Traumatic wounds requiring tissue adhesive closure	4	Wound care and closure using tissue adhesive	0
Bridging	Anaphylactic/allergic reactions	6	IV access and fluids, corticosteroids, antihistamines, epinephrine (IM and/or nebulized), oxygen and monitoring	3
Bridging	Seizures	3	Clinical assessment and observation; wound care or IV access and fluids where required	1
Bridging	Syncope/chest pain	3	Clinical assessment, monitoring and ECG where indicated	1
Bridging	Acute Asthma	1	IV access and fluids, oxygen, ECG monitoring, nebulized epinephrine, ipratropium, salbutamol, IV corticosteroid and magnesium	1
Bridging	Epistaxis with headache and nausea	1	IV access and fluids, IV urapidil and ondansetron	0
Total		18		6

## Data Availability

The data supporting the findings of this study are not publicly available due to data protection and privacy considerations, as the detailed clinical information contained in the dataset may allow for potential re-identification of individual patients despite anonymization. Requests for access to the data may be directed to the corresponding author and will be considered in accordance with applicable data protection requirements.

## Supplementary Materials

The following supporting information can be downloaded at: https://www.mdpi.com/article/10.3390/healthcare14183095/s1, Table S1: Final classification of all evaluated diagnostic and therapeutic measures following the modified Delphi process. The table reports the round in which consensus was reached and the corresponding consensus agreement (n/N and %). For rounds 1 and 2, agreement represents the proportion of participating experts independently supporting the final classification. In round 3, agreement represents the proportion of experts supporting the final classification during the non-anonymous consensus workshop. Green indicates measures suitable for telemedicine-only management (full delegation), yellow indicates measures suitable for bridging treatment (temporary delegation), and red indicates measures requiring direct physician management (no delegation).

## Abbreviations

The following abbreviations are used in this manuscript:

MGE	Mass Gathering Event
MGM	Mass Gathering Medicine
EMT-P	Emergency Medical Technician-Professional
EMT-I	Emergency Medical Technician-Intermediate
