# Supplementary material for: From Crowd to Care: Telemedical Delegation in Mass Gathering Medicine and Its Projected Impact on Hospital Transport—A Modified Delphi Study with Retrospective Modelling
Source: Healthcare (Basel). 2026 Sep 20;14(18):3095. doi: 10.3390/healthcare14183095 (PMC13606773; doi:10.3390/healthcare14183095)
Supplement: Supplementary file 1 [file healthcare-14-03095-s001.zip › healthcare-4540522-supplementary.pdf]

Supplementary files

Supplementary Table S1: Final classification of all evaluated diagnostic and therapeutic measures following the modified Delphi process. The table reports the round in which consensus was reached and the corresponding consensus agreement (n/N and %). For rounds 1 and 2, agreement represents the proportion of participating experts independently supporting the final classification. In round 3, agreement represents the proportion of experts supporting the final classification during the non-anonymous consensus workshop. Green indicates measures suitable for telemedicine-only management (full delegation), yellow indicates measures suitable for bridging treatment (temporary delegation), and red indicates measures requiring direct physician management (no delegation).

Category	Diagnostic and therapeutic measure	Consensus round	Consensus agreement, n/N	Consensus agreement, %
Abdomen	Establish intravenous access	1	4/5	80%
	Assess patient posture (guarding posture / restlessness)	1	5/5	100%
	Hyoscine butylbromide (IV)	2	4/5	80%
	Hyoscine butylbromide (oral)	1	5/5	100%
	Diclofenac (oral)	1	5/5	100%
	Esketamine + midazolam (IV)	2	4/5	80%
	Fentanyl (IV)	3	4/4	100%
	Focused abdominal examination (palpation for areas of tenderness)	1	5/5	100%
	Ibuprofen (oral)	1	5/5	100%
	Loperamide (oral)	1	5/5	100%
	Knee support (knee roll)	1	5/5	100%
	Oral magnesium effervescent tablets	1	5/5	100%
	Metoclopramide (oral)	1	5/5	100%
	Morphine (IV)	3	4/4	100%
	Nalbuphine (IV)	2	4/5	80%
	Metamizole (short IV infusion)	1	4/5	80%
	Metamizole (oral)	1	5/5	100%
	Ondansetron (oral)	1	5/5	100%
	Pantoprazole (oral)	1	5/5	100%
	Acetaminophen (IV)	1	4/5	80%
	Encourage oral fluid intake (water or tea) (suspected urinary tract infection)	1	5/5	100%
	Piritramide (IV)	3	4/4	100%
	Oxygen administration	1	5/5	100%
	Assess for guarding and palpable masses	2	5/5	100%
	Intravenous fluid resuscitation with balanced crystalloid solution	1	4/5	80%
	Dimenhydrinate (oral)	1	5/5	100%
Allergy	Nebulized epinephrine	2	4/5	80%
	Diluted epinephrine (1:1000) (IV)	3	4/4	100%
	Establish intravenous access	1	4/5	80%
	Dexpanthenol ointment (topical)	1	5/5	100%
	Assess the size and extent of the lesion	1	5/5	100%
	Assess the lesion for increased warmth	1	5/5	100%
	Assess for signs of infection	1	5/5	100%
	Cetirizine (oral)	1	5/5	100%

	Wound disinfection with octenidine solution	1	5/5	100%
	Dimetindene gel (topical)	1	5/5	100%
	Dimetindene (oral drops)	1	5/5	100%
	Intravenous H1 antihistamine (e.g. dimetindene)	2	4/5	80%
	Intravenous H2 antihistamine (e.g. cimetidine)	2	4/5	80%
	Hydrocortisone ointment (topical)	1	5/5	100%
	Ibuprofen (oral)	1	5/5	100%
	Ipratropium bromide (inhaled)	2	5/5	100%
	Apply local cooling	1	5/5	100%
	Magnesium (IV)	3	4/4	100%
	Mark the lesion margins to monitor progression	1	5/5	100%
	Pantoprazole (oral)	1	5/5	100%
	Prednisolone or equivalent corticosteroid (IV), depending on availability	2	4/5	80%
	Povidone-iodine ointment (topical)	1	5/5	100%
	Prednisone suppository	1	4/5	80%
	Ethacridine lactate ointment (topical antiseptic)	1	5/5	100%
	Salbutamol (inhaled)	2	5/5	100%
	Apply dressing, supportive bandage, or compression bandage	1	5/5	100%
	Intravenous fluid resuscitation with balanced crystalloid solution	2	5/5	100%
Surgery	Assess tetanus immunization status, previous injuries, prior surgeries, and relevant medical history	1	5/5	100%
	Establish intravenous access	1	4/5	80%
	Dexpanthenol ointment (topical)	1	5/5	100%
	Assess for signs of deep vein thrombosis	1	5/5	100%
	Cetirizine (oral)	1	5/5	100%
	Disinfect the wound with octenidine solution	1	5/5	100%
	Remove small foreign bodies (e.g. splinters) from the wound using gauze and forceps	1	4/5	80%
	Dimetindene gel (topical)	1	5/5	100%
	Fentanyl (IV)	3	4/4	100%
	Silver sulfadiazine cream (topical)	1	5/5	100%
	Elevate the affected limb	1	5/5	100%
	Ibuprofen (oral)	1	5/5	100%
	Inspect the eyes	1	5/5	100%
	Inspect the wound	1	5/5	100%
	Inspect the oral cavity and oropharynx	1	5/5	100%
	Apply local cooling	1	5/5	100%
	Oral magnesium effervescent tablets	1	5/5	100%
	Morphine (IV)	3	4/4	100%
	Nalbuphine (IV)	3	4/4	100%
	Metamizole (short IV infusion)	1	4/5	80%
	Pantoprazole (oral)	1	5/5	100%
	Acetaminophen (oral)	1	5/5	100%

	Encourage oral fluid intake (particularly in patients with a foreign body sensation in the esophagus)	1	5/5	100%
	Piritramide (IV)	3	4/4	100%
	Povidone-iodine (topical)	1	5/5	100%
	Cleanse/irrigate the wound or eyes with normal saline or balanced electrolyte solution	1	5/5	100%
	Immobilize or splint the affected limb	1	5/5	100%
	Assess for signs of fracture	1	5/5	100%
	Assess range of motion	1	5/5	100%
	Assess visual acuity and visual impairment	1	5/5	100%
	Close the wound with tissue adhesive	3	4/4	100%
	Close the wound with adhesive wound closure strips	1	5/5	100%
	Topical diclofenac gel	1	5/5	100%
	Intravenous fluid resuscitation with balanced crystalloid solution	1	4/5	80%
	Apply a wound dressing	1	5/5	100%
Diagnostics	Percuss the costovertebral angles	1	5/5	100%
	Palpate the cervical and submandibular lymph nodes	2	4/5	80%
	Record a single-lead ECG	1	5/5	100%
	Monitor the patient in the on-site medical unit for 30 minutes, followed by discharge back to the festival grounds	1	5/5	100%
	Apply the OPQRST assessment	1	5/5	100%
	Apply the SAMPLER history	1	5/5	100%
	Apply the xABCDE approach	1	5/5	100%
	Auscultate the abdomen	1	4/5	80%
	Auscultate the heart	1	4/5	80%
	Auscultate the lungs	1	5/5	100%
	Assess respiratory rate and breathing pattern (eupnea, dyspnea, abnormal breathing)	1	5/5	100%
	Assess the pulse rate and quality	1	5/5	100%
	Assess the mucous membranes	1	5/5	100%
	Perform a general physical examination	1	5/5	100%
	Perform pulse oximetry	1	5/5	100%
	Discharge the patient back to the festival grounds	1	5/5	100%
	Perform a focused neurological examination (including GCS, pupillary response and level of consciousness)	1	5/5	100%
	Position the patient according to the clinical situation or for comfort	1	5/5	100%
	Measure body temperature	1	5/5	100%
	Measure blood pressure	1	5/5	100%
	Measure blood glucose	1	5/5	100%
	Palpate the abdomen	2	5/5	100%
	Observe and monitor the patient	1	5/5	100%
	Percuss the abdomen	1	4/5	80%
	Percuss the lungs	2	4/5	80%
	Arrange hospital transport	1	4/5	80%
	Document refusal of transport	1	4/5	80%

	Assess and document skin color	1	5/5	100%
	Refer the patient to a general practitioner, out-of-hours primary care service / urgent care, or specialist for further evaluation and treatment	1	5/5	100%
	Obtain a focused medical history according to the presenting complaint	1	5/5	100%
Substance use	Conduct a structured suicide risk assessment in patients with suspected polysubstance intoxication	1	4/5	80%
	Establish intravenous access	1	4/5	80%
	Flumazenil (IV)	2	4/5	80%
	Naloxone (IV)	2	4/5	80%
	Examine for injection sites and other signs of drug use	1	5/5	100%
	Assess for injuries	1	5/5	100%
	Lorazepam (oral)	1	4/5	80%
	Intravenous fluid resuscitation with balanced crystalloid solution	1	4/5	80%
	Dimenhydrinate (oral)	1	5/5	100%
Headache	Inspect and palpate the head	1	5/5	100%
	Establish intravenous access	1	4/5	80%
	Ibuprofen (oral)	1	5/5	100%
	Apply local cooling to areas of contusion	1	5/5	100%
	Oral magnesium effervescent tablets	1	5/5	100%
	Perform a focused neurological examination (BEFAST, orientation, level of consciousness)	1	5/5	100%
	Metamizole (short IV infusion)	1	4/5	80%
	Metamizole (oral)	1	5/5	100%
	Ondansetron (IV)	1	4/5	80%
	Acetaminophen (IV)	1	4/5	80%
	Acetaminophen (oral)	1	5/5	100%
	Assess for photophobia	1	5/5	100%
	Assess for signs of meningeal irritation	1	5/5	100%
	Urapidil (IV)	3	4/4	100%
	Intravenous fluid resuscitation with balanced crystalloid solution	1	4/5	80%
	Dimenhydrinate (oral)	1	5/5	100%
	Provide wound care	1	5/5	100%
Circulation	Record a 12-lead ECG	1	5/5	100%
	Establish intravenous access	1	4/5	80%
	Aspirin (IV)	3	4/4	100%
	Reassure and calm the patient	1	5/5	100%
	Diclofenac (oral)	1	5/5	100%
	Fentanyl (IV)	3	4/4	100%
	Heparin (IV)	3	4/4	100%
	Ibuprofen (oral)	1	5/5	100%
	Apply local cooling to the neck in case of epistaxis	1	5/5	100%
	Morphine (IV)	3	4/4	100%
	Perform nasal packing	1	5/5	100%
	Glyceryl trinitrate sublingual spray	3	4/4	100%
	Metamizole (short IV infusion)	1	4/5	80%

	Ondansetron (IV)	1	4/5	80%
	Acetaminophen (IV)	1	4/5	80%
	Acetaminophen (oral)	1	5/5	100%
	Metamizole (oral)	1	5/5	100%
	Urapidil (IV)	3	4/4	100%
	Intravenous fluid resuscitation with balanced crystalloid solution	1	4/5	80%
	Provide wound care	1	5/5	100%
Critical	Percuss the spine	3	4/4	100%
	Palpate the chest	1	4/5	80%
	Epinephrine (IM), including repeated administration at intervals of a few minutes if required	2	5/5	100%
	Diluted epinephrine (1:1000) (IV)	3	4/4	100%
	Nebulized undiluted epinephrine	2	5/5	100%
	Record a 12-lead ECG	3	4/4	100%
	Establish intravenous access	3	4/4	100%
	Aspirin (IV)	2	4/5	80%
	Insert an oropharyngeal airway (Guedel airway)	1	5/5	100%
	Insert a laryngeal mask airway or laryngeal tube	1	5/5	100%
	Insert a nasopharyngeal airway (Wendl airway)	1	4/5	80%
	Esketamine (IV)	2	4/5	80%
	Establish intraosseous access	1	5/5	100%
	Fentanyl (IV)	3	4/4	100%
	Intravenous H1 antihistamine (e.g. dimetindene)	3	4/4	100%
	Intravenous H2 receptor antagonist (e.g. cimetidine)	3	4/4	100%
	Heparin (IV)	2	4/5	80%
	Ipratropium bromide (inhaled)	3	4/4	100%
	Magnesium (IV)	2	4/5	80%
	Morphine (IV)	3	4/4	100%
	Midazolam (IV)	2	4/5	80%
	Assess for a postictal state (differential diagnosis: seizure vs. convulsive syncope)	1	4/5	80%
	Nalbuphine (IV)	3	4/4	100%
	Glyceryl trinitrate sublingual spray	2	4/5	80%
	Piritramide (IV)	3	4/4	100%
	Prednisolone or equivalent corticosteroid (IV), depending on availability	3	4/4	100%
	Assess for jugular venous distension	2	4/5	80%
	Assess for abnormal chest wall movement	2	4/5	80%
	Assess for tongue bite	3	4/4	100%
	Assess for cyanosis	3	4/4	100%
	Salbutamol (inhaled)	3	4/4	100%
	Administer oxygen	3	4/4	100%
	Assess for additional injuries	1	4/5	80%
	Assess the airway for patency and signs of swelling	3	4/4	100%
	Urapidil (IV)	3	4/4	100%
	Intravenous fluid resuscitation with balanced crystalloid solution	3	4/4	100%
